# The sepsis model: an emerging hypothesis for the lethality of inhalation anthrax

**DOI:** 10.1111/jcmm.12075

**Published:** 2013-06-07

**Authors:** Kenneth Mark Coggeshall, Florea Lupu, Jimmy Ballard, Jordan P Metcalf, Judith A James, Darise Farris, Shinichiro Kurosawa

**Affiliations:** a Immunobiology and Cancer Program, Oklahoma Medical Research FoundationOklahoma City, OK, USA; b Cardiovascular Biology Program, Oklahoma Medical Research FoundationOklahoma City, OK, USA; c The Department of Microbiology, University of Oklahoma Health Sciences CenterOklahoma City, OK, USA; d The Department of Medicine, University of Oklahoma Health Sciences CenterOklahoma City, OK, USA; e Arthritis and Clinical Immunology Program, Oklahoma Medical Research FoundationOklahoma City, OK, USA; f Department of Pathology & Laboratory Medicine, Boston University School of MedicineBoston, MA, USA

**Keywords:** Sepsis, anthrax, lethal factor, oedema factor, disseminated intravascular coagulation, Gram-positive

## Abstract

Inhalation anthrax is often described as a toxin-mediated disease. However, the toxaemia model does not account for the high mortality of inhalation anthrax relative to other forms of the disease or for the pathology present in inhalation anthrax. Patients with inhalation anthrax consistently show extreme bacteraemia and, in contrast to animals challenged with toxin, signs of sepsis. Rather than toxaemia, we propose that death in inhalation anthrax results from an overwhelming bacteraemia that leads to severe sepsis. According to our model, the central role of anthrax toxin is to permit the vegetative bacteria to escape immune detection. Other forms of *B. anthracis* infection have lower mortality because their overt symptoms early in the course of disease cause patients to seek medical care at a time when the infection and its sequelae can still be reversed by antibiotics. Thus, the sepsis model explains key features of inhalation anthrax and may offer a more complete understanding of disease pathology for researchers as well as those involved in the care of patients.

## Introduction

As a result of letter mailings in 2011, 11 individuals were diagnosed with inhalation anthrax, of whom only six survived 1. The incident caused terror and panic as many thousands in the targeted regions expressed concerns of having been exposed 2–4. The event brought an intense focus on the pathology of inhalation anthrax and its causative agent, *Bacillus anthracis*. Unlike cutaneous or gastrointestinal anthrax, which are rarely lethal, inhalation anthrax has a mortality rate of 50–90% 5. The reasons for the high mortality of inhalation anthrax are perplexing. For years, mortality in anthrax infections has been attributed to toxaemia 6, 7, largely because the toxin is able to cause the death of experimental animals (discussed below) and immunity to the toxin protects from infections 8, 9. However, the toxaemia model for the high mortality of inhalation anthrax does not account for the lower mortality of cutaneous and gastrointestinal forms of the disease, where the disease is caused by the same organism and circulating toxins are also present 10. Furthermore, the pathology in toxin-challenged animals does not resemble the pathology in *B. anthracis*-challenged animals. Therefore, although widely accepted, a growing body of evidence indicates that the toxaemia model of inhalation anthrax is incomplete. Emerging data indicate an alternative view: that *B. anthracis* uses the toxins to suppress host immunity, allowing the organism to proliferate to high numbers and causing death by septic shock.

## Discussion of the evidence

### The anthrax toxins and toxin receptors

The anthrax bacillus produces two exotoxins that are encoded on a plasmid, pOX1, producing three protein products. The first protein is protective antigen (PA), an 83-kD protein that is common between the two exotoxins and confers receptor-binding properties. PA binds to two receptors, tumour endothelial marker 8 (TEM8) and capillary morphogenesis protein 2 (CMG2). The CMG2 receptor for PA is the major mouse PA receptor 11 and is widely expressed on a variety of cells and tissues 12 while the TEM8 receptors are expressed in epithelium 13, 14 and up-regulated in endothelium in response to inflammatory stimuli 15. PA associates with two other proteins encoded on pOX1, lethal factor and oedema factor, to become lethal toxin (LT) and oedema toxin (ET) respectively. Remarkably, although toxins were lethal in myeloid-restricted *CMG2*^*−/−*^ mice, these mice were resistant to infection with toxigenic and encapsulated *B. anthracis* 16. Similarly, the bacteraemia that normally accompanies *B. anthracis* infections did not occur in *CMG2*^*−/−*^ mice. As *CMG2*^*−/−*^ and myeloid-restricted *CMG2*^*−/−*^ mice are resistant to toxin-induced immune suppression, these findings suggest that anthrax toxin plays a key role in supporting the development of bacteraemia by suppressing a key feature(s) of the immune responses to the bacteria. This view contrasts with results of experiments using toxin-producing and toxin-lacking *B. anthracis* spores and a macrophage cell line, J774 17. These data showed that J774 was able to produce inflammatory cytokines regardless of the ability of the vegetative bacteria to produce toxin, suggesting that toxins do not suppress this feature of inflammation. In *B. anthracis*-infected guinea pigs, PA reaches maximum levels of <2 μg/ml of blood while rabbits can achieve as much as 100 μg/ml 18. A correlation between bacteraemia and PA levels has been shown 19, consistent with the notion that toxin is needed for *B. anthracis* to escape immune detection in the host.

### Clinicopathological features of human inhalation anthrax

Although inhalation anthrax is an exceedingly rare occurrence, there are two well-documented major outbreaks in recent history. The first occurred in Sverdlovsk, USSR in 1979 and was attributed many years later to the accidental release of anthrax spores from a nearby military facility 20. An analysis of available data suggested that there were about 250 total cases with 100 deaths 21. Pathological studies of fixed and preserved samples of 41 fatally infected individuals from the Sverdlovsk release 22 indicated overwhelming bacteraemia, a hallmark of late-stage inhalation anthrax in human beings 5 and animal models 23. Bacilli were found in most organs, particularly the lung, mediastinal lymph node and the spleen. Acute bronchopneumonia and fibrin-rich lung oedema were present in half of the examined cases and involved all five lobes of the lung. The pathogen was more prominent in the vascular and interstitial spaces than in the alveoli, suggesting terminal seeding of the lung from the vasculature. In addition to bacteraemia, the samples showed several haemorrhagic features. A microscopic analysis revealed vasculitis and fibrin deposition with high- and low-pressure haemorrhages 22. One-third of the cases showed early renal acute tubular necrosis. Although not a consensus opinion, one author of the study, who had participated in treating the patients, suggested that the haemorrhagic features were the result of disseminated intravascular coagulation (DIC) 22, as the patients also showed decreased fibrinogen and increased bleeding times prior to their deaths. Importantly, haemorrhagic diathesis was present pathologically in over 70% of these cases 22.

The second well-documented inhalation anthrax outbreak occurred in late 2001. Five letters containing the spores of *B. anthracis* passed through postal facilities in the District of Columbia, New Jersey and Florida. Initially, ten patients appeared from sites through which the letters had passed 24. Pathological studies of the patients who died in this U.S. exposure 1, 25 showed pathology similar to that described for the Sverdlovsk outbreak. Bacterial antigens were prominent in all tissues except the lung, and patients had elevated neutrophils, including immature forms (bands). Haemorrhagic features were also seen, including serosanguinous pleural effusions, haemorrhage and oedema in the lung parenchyma, and varying degrees of haemorrhage and/or necrosis in the spleen, intestine and mediastinal lymph nodes.

Although even more rare, there have been case reports of disease in which the infectious agent was the *B. anthracis*-related species *B. cereus* harbouring the toxin-encoding pOX1 plasmid 26–28. The most detailed report 28 described a single 39-year-old male patient who presented to the emergency room with shortness of breath, hypotension, tachycardia, multicentric pneumonia and pleural effusions. The patient died 72 hrs after initial presentation, despite the identification of a *Bacillus* infection less than 10 hrs after presentation and administration of intensive antibiotic therapy. The pathology observed in this case was largely consistent with that of inhalation anthrax. Laboratory tests revealed elevated blood levels of neutrophils (21,000/μl) and elevated plasma D-dimers (1574 ng/ml) indicative of DIC. Both lungs showed serosanguinous fluid, and unlike inhalation anthrax caused by *B. anthracis*, the intact pathogen was prominent in the alveolar space. All organs except skin were positive for *B. cereus* by chemical staining. Microscopic examination of tissues showed fibrin deposition, indications of ischaemia and areas of necrosis, particularly in the kidneys.

Together, these observations establish several common features of infections with pOX1-harbouring *Bacillus* species and provide clues to the pathophysiology of lethal inhalation anthrax. First, both inhalation anthrax and respiratory *B. cereus* infection lead to widespread and extraordinary bacteraemia, as well as clear signs of inflammation and hypotension, accompanied by tachycardia. Late stages of infection are marked by oedema, particularly in the lung, fibrin deposition and thrombosis, particularly in the kidney, haemorrhage associated with vasculitis, and necrosis in multiple tissues, particularly the kidneys.

### Pathological features of animal models challenged with toxin

If toxin challenge is an accurate model for the lethality caused by inhalation anthrax, then death caused by toxin challenge and death caused by inhalation anthrax should share similar pathological features. For the most sensitive mouse strains, Balb/c 29 or A/J 30, 100 μg of LT was lethal in about 60% of animals within 3 days regardless of the route of administration (intraperitoneal or intravenous injection) 29. LT challenge resulted in mild apoptosis of myeloid and lymphoid cells in the spleen and necrosis in the liver. Signs of haemorrhage appeared in the spleen but resolved prior to death, and only mild haemorrhage occurred in the liver with no deposition of fibrin. No kidney pathology or capillary thrombosis was observed in mice challenged with LT. ET is more potent than LT in mice; 37.5 μg ET was lethal in 50% of mice in less than 3 days 31. ET-challenged mice showed fluid accumulation in the intestine and necrosis in several organs, particularly the heart. In dogs, intravenous administration of 8.4 μg/kg LT or 205 μg/kg ET was 50% lethal at 24 hrs. As in mice and rats, each toxin induced hypotension and tachycardia, but unlike mice and rats each toxin caused kidney injury, demonstrated by increases in blood urea nitrogen and creatinine 32. In combination, the pathology produced by the two toxins appeared to be additive rather than synergistic 33. In contrast to inhalation anthrax infections, there were no obvious changes in pleural or peritoneal fluid levels and, importantly, no signs of inflammation in animals challenged with either or both toxins.

These studies in various animal models reveal that toxin challenge produces hypotension, a feature common to the human cases of inhalation anthrax. The mechanism of toxin-induced hypotension remains unclear, but could involve vasodilation or LT-induced apoptosis of vascular endothelial cells 34. However, there are few other similarities between the pathology of toxin challenge and that of inhalation anthrax infection, and the key features of inhalation anthrax are largely absent in animals challenged with toxin alone. In toxin-challenged animals, there is a marked absence of inflammation and limited production of inflammatory cytokines, although IL-1β is elevated with infection and with LT challenge probably by activation of Nalp1b and caspase-1 35. Although these observations do not specifically exclude the toxaemia model, they strongly challenge the notion that the pathology accompanying inhalation anthrax is directly due to the toxins.

This conclusion is supported by the relative low potency of LT and ET compared with other bacterial toxins. As discussed above, the lethal dose of LT can range from 20 μg to more than 100 μg per mouse. While more lethal than LT, ET nevertheless exhibits an LD_50_ no higher than 25–50 μg in a ∼20 g mouse. This dose of toxin is more than 1000-fold higher than that needed for lethality by other bacterial toxins like diphtheria, pertussis or cholera 36. On the basis of these lines of evidence, we conclude that mortality in inhalation anthrax is not solely due to toxaemia and suggest that other features of the bacteria contribute to the pathology.

### Sepsis in anthrax infections

The rapid development of sepsis is well documented in cases of injectional anthrax, in which the blood is directly infected with *B. anthracis via* intravenous injection with illicit drugs. At least 47 cases of injectional anthrax, including 13 deaths, were confirmed in drug addicts in Norway 37, Scotland 38 and Germany 39. These patients rapidly developed all the landmark features of sepsis (sustained fever, decreased or elevated white blood cell count, anaemia, thrombocytopaenia) although the severity of the pathology might in part be due to drug use by the patients. When infection was not detected and treated expeditiously, the patients developed septic shock followed by multiple organ failure characterized by elevated liver enzymes, acute renal insufficiency, DIC and massive disseminated bleeding 40. Because direct infection of blood in injectional anthrax leads to severe and rapidly lethal sepsis, it is likely that the remarkable bacteraemia observed in inhalation anthrax can also cause sepsis.

In addition, our recent studies demonstrate that infusion of live *B. anthracis* causes sepsis in non-human primates 41. These animals exhibited systemic inflammation typical of sepsis 42, as well as consumptive coagulopathy indicated by decreased fibrinogen and platelets, prolonged clotting times, and elevated fibrinogen degradation products. Such abnormalities in blood coagulation are characteristic of DIC, which is strongly associated with clinical illnesses such as sepsis 43. Importantly, these changes do not occur in animal models challenged with toxin alone. Furthermore, the pathology of *B. anthracis*-challenged animals is remarkably similar to that described for the 2001 letter attack victims, who also showed coagulopathy 25, 44.

Sepsis exhibits a variable set of clinical features 45 but is usually described as systemic inflammation caused by an infectious agent. Clinical features include core temperatures >38.3°C or <36°C, elevated heart rate, altered mental status and significant oedema (>20 mls/kg in 24 hrs) 46. Inflammation is indicated by elevated or decreased blood neutrophils, increased levels of immature neutrophils, thrombocytopaenia and elevated circulating C-reactive protein. Haemodynamic abnormalities include decreased arterial pressure and prolonged measurements of blood clotting (prothrombin time and partial thromboplastin time). When accompanied by evidence of hypoperfusion or dysfunction of at least one organ system, the situation evolves to severe sepsis and organ failure. Organ dysfunction features include decreased blood oxygenation, decreased urine output, and elevated metabolite levels such as bilirubin and creatinine. Finally, if severe sepsis is accompanied by hypotension (<90 mmHg or a reduction of ≥40 mmHg from baseline) or need for vasopressors, despite adequate fluid resuscitation, the disease has progressed to ‘septic shock’. These features are consistent with the pathology seen in victims of the letter attacks and of the Sverdlovsk spore release, while most of these features are absent in toxin-challenged animals.

Sepsis is always accompanied by elevated proinflammatory cytokines 47, which contributes to the activation of blood coagulation through tissue factor expression 48 and to haemodynamic instability 49. The dysregulated coagulation reported in inhalation anthrax is common in other forms of sepsis. For example, lipopolysaccharide (LPS) directly elicits increased expression of tissue factor on monocytes 50. LPS alone can account for the systemic inflammation and coagulopathy in Gram-negative sepsis as challenge of animals 51 or human beings 52, 53 with LPS induces a clinical inflammatory response that resembles Gram-negative sepsis.

How Gram-positive bacteria like *B. anthracis* cause systemic inflammation remains unclear. Live *B. anthracis* bacteria harbour several proinflammatory agents that can stimulate inflammation *in vitro* 54–57. We 58–60 and others 61, 62 have provided evidence that the peptidoglycan (PGN) component of the bacterial cell wall induces proinflammatory cytokine production in innate immune cells. *In vivo*, PGN stimulates proinflammatory cytokines in rats 63 but *in vitro* mouse macrophages fail to respond 59. A recent review, noting the ability of *B. anthracis* cell wall preparations to induce inflammation and shock in rats 63, suggested *B. anthracis* PGN as the inflammatory agent in injectional and inhalation anthrax 64. Our recent work shows that the proinflammatory response in human cells is due to the formation of PGN-anti-PGN immune complexes that bind to IgG Fc receptors expressed on innate immune cells 65. After these immune complexes undergo phagocytosis and lysosomal hydrolysis, their degradation products are available for detection by cytoplasmic nucleotide oligomerization domain-containing (NOD) receptors 65. NOD receptors induce proinflammatory cytokines through the activation of NFκB 66. Recent but unpublished studies by our laboratories showed that the PGN-anti-PGN immune complexes also activate primate platelets that, unique in the animal kingdom, also express Fc receptors. Thus, the engagement of Fcgamma receptors by PGN-anti-PGN immune complexes is one possible route for *B. anthracis* to induce systemic inflammation in human beings and other primates.

## Summary

**Fig. 1:**
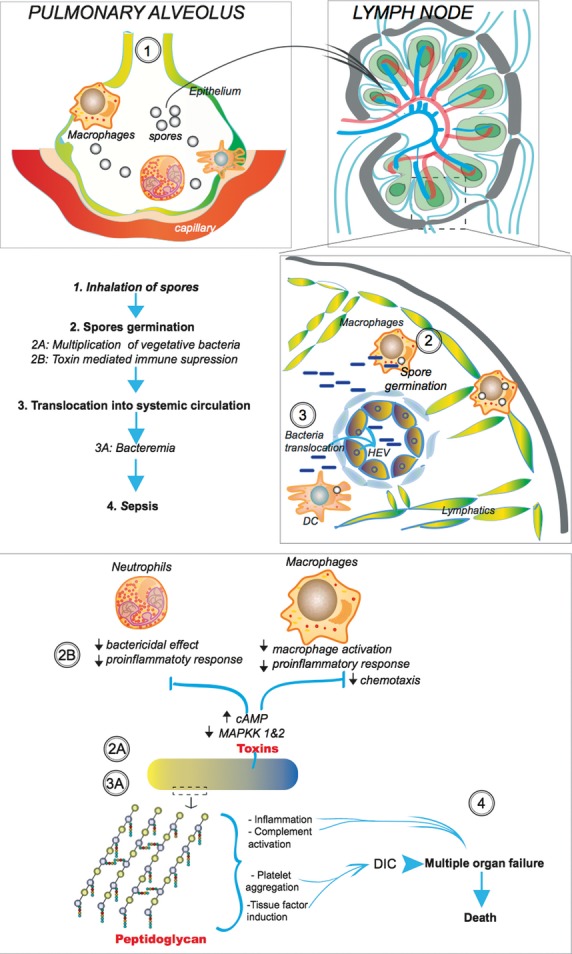
The sepsis model. (1) Spores are inhaled (top left) and move from the airways into the mediastinal lymph node (top right and middle panels) through an unknown mechanism. (2) Spore germination occurs either within the mediastinum or at a point before entering the organ. The bacteria proliferate and produce toxin which suppresses the immune response and enables the bacteria to overwhelm innate immunity. (3) The bacteria enter the blood circulation where continued bacterial proliferation eventually yields to extreme bacteraemia and eventual sepsis. (4) We hypothesize that the peptidoglycan present in the vegetative bacteria and shed during bacterial replication causes systemic inflammation, complement activation and platelet aggregation. These sepsis-associated events contribute to disseminated intravascular coagulopathy (DIC), organ failure, and eventual death of the patient.

Like tetanus, diphtheria and botulism, anthrax is now considered to be a toxin-mediated disease. However, a growing body of evidence supports the conclusion that anthrax toxins alone are not the sole cause of high mortality in inhalation anthrax. In contrast, remarkably similar pathological features, including several hallmarks of sepsis, were observed in non-human primates challenged with *B. anthracis* and in inhalation anthrax patients from both Sverdlovsk and the 2001 letter attacks in the United States. In the sepsis model, outlined in Figure 1, we propose that inhalation anthrax begins with the introduction of a sufficient number of spores such that the mediastinal lymph nodes become infected with vegetative bacteria. In the lymph nodes, the struggle between host immunity and bacteraemia begins: vegetative *B. anthracis* produces virulence factors including toxins that suppress immune detection and/or the host immune response 16. At this stage, antibiotics or anti-toxin antibodies could clear the bacteria, but because the patient feels only mildly sick 5 they delay medical attention. Indeed, in cutaneous or gastrointestinal anthrax, which cause overt symptoms of disease, patients seek medical attention promptly and antibiotics clear the infection 67. This notion is amply illustrated in a recent case of apparent gastrointestinal anthrax in the United States 68. A woman seeking medical attention complained of a variety of non-descript issues but included her recent development of nausea, vomiting and decreased food intake. Upon admission to the hospital, her clinical features included prolonged clotting times, thrombocytopaenia, and Gram-positive rods in blood cultures, all indicating sepsis. These rather extreme symptoms likely caused the patient to obtain medical advice and resulted in her survival after extreme medical intervention. Without prompt and proper intervention, the balance between immunity and infection will eventually tip in favour of the proliferating bacteria. The vegetative bacteria enter blood circulation and overwhelming bacteraemia occurs. At this point, the patient is sufficiently ill to seek medical attention, but antibiotics or anti-toxin antibodies are unable to prevent imminent death from the already advanced systemic inflammation and coagulopathy.

It should be emphasized that the sepsis model we describe here invokes an important role for anthrax toxins in the progression and ultimate lethality of inhalation anthrax. We propose that by suppressing the immune response, anthrax toxins play a central and necessary role in the development of bacteraemia and lethal sepsis. It is also likely that the anthrax toxins contribute to the pathology caused by the pronounced bacteraemia, especially late in the disease when toxins are at their highest level. A central role for anthrax toxins in disease development explains why vaccination against toxin can protect human beings from inhalation anthrax: without the immune suppression caused by anthrax toxins, the vegetative bacilli are effectively cleared by the immune system long before they become a systemic challenge. The model also explains why treatments such as Raxibacumab, the recently FDA-approved monoclonal antibody targeting PA, can protect animals 69. However, according to our model, Raxibacumab prevents anthrax toxins from incapacitating innate immune cells so that they are free to kill the microbe. Thus, the time window for efficacious treatment by Raxibacumab overlaps that for antibiotics, which we believe fail because patients do not yet know they have the disease.

If the sepsis model of inhalation anthrax is correct, our understanding of the basic biology of inhalation anthrax is confined and incomplete by the toxin-centred focus of current studies. More importantly, the toxin-centric approach in the study of inhalation anthrax pathology will do little to save patients with inhalation anthrax. Rather, we believe the research focus should be on how Gram-positive bacteria escape human immune detection, how they establish bacteraemic conditions in human beings, and how they cause systemic inflammation in human beings.
